# Diagnosis, Pathology, and Treatment of Diseases of the Chest

**Published:** 1847-04

**Authors:** 


ARTICLE VII.

*The Diagnosis, Pathology, and Treatment of Diseases of the Chest:* By W. W. Gerhard, M. D., Lecturer on Clinical Medicine to the University of Pennsylvania; one of the Physicians to the Pennsylvania Hospital, etc. Second Edition, revised and enlarged. Philadelphia: Ed. Barrington & Geo. Haswell. 1846. pp. 288.

We are gratified to see a second edition of this deservedly popular work. The great importance of the class of diseases of which it treats, and the acknowledged ability and qualifications of its author, to do it ample justice, are good reasons why it should be in the library of every physician in the country. E.

